# A Promoter Polymorphism (rs17222919, −1316T/G) of ALOX5AP Gene Is Associated with Decreased Risk of Ischemic Stroke in Two Independent Chinese Populations

**DOI:** 10.1371/journal.pone.0122393

**Published:** 2015-03-27

**Authors:** Yujia Fan, Hui Chen, Aifan Li, Yunshu Shi, Yuchao Zhang, Qingchuan Feng, Yan Sun, Hong Zheng, Ying He

**Affiliations:** 1 Department of Cell Biology and Medical Genetics, Basic Medical College of Zhengzhou University, Zhengzhou, China; 2 Department of Neurology, the First People Hospital of Zhengzhou, Zhengzhou, China; Huashan Hospital, Fudan University, CHINA

## Abstract

No coding sequence variants of the gene encoding 5-lipoxygenase-activating protein (*ALOX5AP*) leading to amino acid substitutions have been identified. Therefore, variants in the *ALOX5AP* promoter region have received attention recently. The purpose of this study was to explore whether the promoter polymorphism rs17222919 is involved in the etiology of ischemic stroke (IS) in the Chinese Han population. We investigated the rs17222919 polymorphism by TaqMan genotyping in two independent Chinese Han samples: the first comprised 910 IS patients and 925 healthy inhabitants from the northern Henan Province, while the second included 1003 IS patients and 889 healthy controls from the southern Henan Province. Functional characterization of rs17222919 was performed by an *in vitro* luciferase assay. After adjusting for conventional risk factors, the G allele frequencies in the IS groups were significantly lower than that in the control groups of the two independent Chinese cohorts (19.0% vs. 22.9%, *P* = 0.004, odds ratio (OR) = 0.792, 95% confidence interval (CI) = 0.675–0.929; 18.8% vs. 22.9%, *P* = 0.002, OR = 0.782, 95% CI = 0.668–0.915, respectively). This was also observed in the large-artery atherosclerosis (LAA) and stroke of other undetermined etiology (SUE) subtypes (*P* = 0.019, OR = 0.815, 95% CI = 0.687–0.967; *P* = 0.021, OR = 0.815, 95% CI = 0.685–0.970, respectively). Additionally, the TG genotype and G allele frequencies were significantly lower in the IS compared with the control group in two female cohorts (P<0.05). Finally, the *in vitro* luciferase assay demonstrated that the G allele has a significantly lower transcription activity than the T allele (*P* = 0.031). Our study provides evidence that the promoter single nucleotide polymorphism (SNP) rs17222919 is a potential genetic protective factor for IS in the Chinese Han population.

## Introduction

Ischemic stroke (IS) is a complex multifactorial disorder characterized by stenosis or occlusion of the cerebral arteries that is mainly caused by atherosclerotic lesions on the vascular wall [1]. Arterial wall inflammation plays an important role in the pathogenesis of atherosclerosis and ultimately leads to atherosclerotic plaque rupture, fissure, or erosion, which is the major pathological basis of IS[2]. The 5-lipoxygenase activating protein (FLAP), encoded by the *ALOX5AP* gene, is a crucial mediator of the biosynthesis of leukotrienes (LTs) and causes the accumulation of LTs in fatty deposits on the arterial wall [3]. The subsequent collapse of these deposits by the immune system has been implicated in the development of atherosclerosis and an increased risk of IS [4].

Human *ALOX5AP* gene (NT_024524) is located on chromosome 13q12.3 and contains five exons [5]. Helgadottir and colleagues reported that two at-risk haplotypes (HapA and HapB) of *ALOX5AP* gene were associated with myocardial infarction and stroke in the Icelandic population [6]. Subsequently, replications of these findings have been conducted in various populations [7–9]. However, despite accumulating support for an association between *ALOX5AP* variants and cerebral vascular events, the precise mechanism remains unclear. Interestingly, no coding sequence variants of *ALOX5AP* gene leading to amino acid substitutions have been identified [10]. Moreover, previous studies revealed that the *ALOX5AP* gene expression levels and its downstream leukotriene B_4_ (LTB_4_) synthesis activity were greater in IS patients than in controls [11, 12]. We therefore hypothesized that as yet unidentified variants in the promoter region of *ALOX5AP* gene might be associated with the potential risk of IS.

Kim et al [13] previously reported that the *ALOX5AP* promoter single nucleotide polymorphism (SNP) rs17222919 was associated with the development of intracerebral hemorrhage in the Korean population. However, few studies have investigated the association between rs17222919 and susceptibility to IS in Chinese population. Therefore, the present study aimed to investigate the role of rs17222919 polymorphism in IS risk in a northern Henan Han population, to replicate the positive findings in a second cohort from a southern Henan Han population, and to carry out functional characterization of the rs17222919 SNP using an *in vitro* luciferase assay.

## Materials and Methods

### Study populations

A two-stage study design was used to evaluate the *ALOX5AP* promoter SNP rs17222919 in relation to IS risk and then to validate any promising associations in a second independent population. In the initial study, 910 patients with IS (479 men and 431 women, mean age of 56.1±10.6 years) were recruited from two medical centers in Zhengzhou and Xinxiang. The diagnosis of IS was based on a loss of global or focal cerebral function persisting for >24 h with corresponding infarction on brain imaging with a probable vascular cause [14]. Only patients with three subtypes of IS were recruited, including those with large-artery atherosclerosis (LAA), small-artery occlusion lacunar (SAO), and stroke of other undetermined etiology (SUE), according to the Trial of Org 10172 in Acute Stroke Treatment (TOAST) [15]. Brain imaging was carried out by computed tomography and/or magnetic resonance imaging, and ancillary diagnostic investigations and standardized blood tests were also performed. Patients with atrial fibrillation, cerebral hemorrhage, peripheral vascular diseases, or kidney diseases were excluded from the study. The control group consisted of 925 unrelated Henan Han individuals (478 men and 447 women, mean age 55.3±10.3 years), selected from the same demographic area and matched to the cases by age, sex, and residency. All controls were free of cerebrovascular disease, cardiovascular disease, and cancer, hepatic, or renal diseases.

For the purpose of replication, another independent case-control cohort comprising 1003 IS patients and 889 controls were recruited from two medical centers in Nanyang and Xinyang, respectively. The IS diagnostic criteria and control recruitment criteria were identical to those of the first population. There were no overlapping participants between the two populations.

The study protocols were approved by the Ethics Committee on Human Research of Zhengzhou University and informed written consent was obtained from each participant.

### Genotyping of rs17222919 polymorphism

EDTA anti-coagulated venous blood samples were collected from each subject enrolled in the current investigation. Genomic DNA was extracted from the peripheral blood using an AxyPrep Blood Genomic DNA Miniprep Kit (Axygen Biotechnology, Union City, CA, USA) according to the manufacturer’s instructions. For SNP genotyping, TaqMan probes were used to analyze the rs17222919 polymorphism (S1 Fig). The genotype was determined according to the relative fluorescence intensity of the probe detected by the real-time PCR system (ABI PRISM 7500; Applied Biosystems, Foster City, CA, USA). Each PCR reaction system (10 μL) contained 5 μL of 2 × TaqMan Universal PCR Master Mix (Applied Biosystems), 0.2 μL forward primer, 0.2 μL reverse primer, 0.6 μL FAM-labeled Probe, 0.6 μL HEX-labeled Probe, 0.4 μL ROXII, 1.0 μL of DNA template (1–20 ng/μL), and 2 μL ddH_2_O. In each assay, three samples with known genotypes and three no-DNA blank controls were included. The PCR reaction conditions consisted of pre-degeneration for 2 min at 95°C, then denaturation at 95°C for 15 s, and 40 cycles of annealing and extension for 30 s at 60°C. After PCR amplification, sample genotypes were attributed by measuring the allele-specific fluorescence with an ABI Prism 7700 Sequence Detection System using SDS 1.7 software for allele discrimination (Applied Biosystems). To verify the genotyping accuracy, 10% of random samples were sequenced (S2 Fig).

### Promoter–luciferase constructs and luciferase assay

We used a luciferase assay to determine whether rs17222919 modulated the activity of the *ALOX5AP* promoter. *ALOX5AP* promoter fragments containing rs17222919 (331-bp) were amplified by PCR using the primer pair 5′-CGGGGTACCGCAGATGGCAAACCATGAA-3′ (forward) and 5′- GGAAGATCTGGGAAGATATTGCTCCCTCA-3′ (reverse). Next, PCR products were purified and digested using *Kpn* I and *Bgl* II restriction endonucleases. Positive clones were confirmed by DNA sequencing (Gen-Script, Nanjing, China).

For luciferase reporter assays, HEK293 cells were cultured in Dulbecco’s modified Eagle’s medium (Invitrogen, Carlsbad, CA, USA) supplemented with 15% fetal bovine serum (Hyclone, Logan, UT, USA) at 37°C in a humidified incubator containing 5% CO_2_. The HEK293 cells were plated at a density of 1×10^5^ per well in 24-well format, cultured for 48 h, then co-transfected with 80 ng pGL3-promoter construct and 4 ng pRL-TK co-transfector using 1 μL of Lipofectamine 2000 (Invitrogen) according to the manufacturer’s recommendations. Luciferase activity was assayed using the Dual-Luciferase Reporter Assay System (Promega, Madison, WI, USA). Firefly luciferase expression levels were adjusted with reference to Renilla luciferase activity. Three independent experiments were performed for each reporter (S1 Dataset).

### Statistical analysis

Pearson’s chi-squared test was used to test for differences in qualitative variables and genotype/allele frequencies. Differences in quantitative variables between groups were analyzed using the Student’s *t*-test. Hardy-Weinberg equilibrium (HWE) testing was carried out using SHEsis software (http://analysis.bio-x.cn) [16]. Odds ratios (ORs) and 95% confidence intervals (CIs) were obtained to assess the association of rs17222919 with IS risk using Unconditional Univariate logistic regression models. Genotypes were further stratified by sex and subgroups of clinical/pathological variables. Results obtained from luciferase assays were compared using one-way ANOVA analysis of variance for repeated measurements followed by the Bonferroni test. All statistical analysis was performed using the SPSS 17.0 package (SPSS Inc., Chicago, IL, USA). *P* values less than 0.05 (two-tailed) were considered statistically significant.

## Results

### Characteristics of the subjects

Two groups of IS cases and controls were recruited from the Henan Province of China. One group (Population 1) was from the northern Henan Province whereas the other (Population 2) was from the southern Henan Province. Clinical and pathological characteristics of Population 1 and Population 2 were shown in Table 1. Cases and controls were well matched in age and sex (*P*>0.05). Compared with the control groups, IS groups showed higher percentages of hypertension, diabetes mellitus, smokers and consumed alcohol (*P*<0.05). IS patients also had significantly higher total cholesterol and total triglyceride levels than the control subjects (*P*<0.05).

**Table 1:** Characteristics of the study populations.

	Population 1			Population 2		
	Cases(n = 910)	Controls(n = 925)	*P* value	Cases(n = 1003)	Controls(n = 889)	*P* value
Sex(males/females)	479/431	478/447	0.680	542/461	458/431	0.273
Age(mean±SD, years)	56.1±10.6	55.3±10.3	0.408	60.5±7.8	59.6±7.4	0.137
Total cholesterol(mmol/L)	5.05±0.89	4.72±1.04	0.000*	4.76±1.13	4.45±1.21	0.004*
Total triglyceride(mmol/L)	1.81±1.23	1.71±1.04	0.072	1.68±1.15	1.37±1.16	0.005*
Hypertension, (n, %)	201(22.1)	98(10.6)	0.000*	206(20.5)	114(12.8)	0.000*
Diabetes, (n, %)	134(14.7)	26(2.8)	0.000*	176(17.5)	25(2.8)	0.000*
Smokers, (n, %)	112(12.3)	64(6.9)	0.000*	166(16.6)	58(6.5)	0.000*
Alcohol, (n, %)	133(14.6)	77(8.3)	0.000*	168(16.7)	60(6.7)	0.000*

*P < 0.05 denotes statistical significance

### Association analysis and inherited model test

In the two control groups, genotype frequency distributions of rs17222919 were consistent with Hardy-Weinberg equilibrium (HWE) in Population 1 and Population 2 (*P* = 0.947 and *P* = 0.937, respectively).

In Population 1, multivariate logistic regression analysis was used after adjusting the effects of conventional risk factors, and the G allele frequency (19.0%) in the IS group was found to be significantly lower than that (22.9%) in the control group (*P* = 0.004, OR = 0.792, 95% CI = 0.675–0.929). Both the homozygous GG genotype frequency (3.2%) and heterozygote TG genotype frequency (31.6%) in the IS group were significantly lower than those (5.2% and 35.3%, respectively) in the control group (*P* = 0.016, OR = 0.560, 95% CI = 0.348–0.901; *P* = 0.043, OR = 0.817, 95% CI = 0.671–0.994, respectively) (Table 2).

**Table 2:** Genotype and allelic distribution of rs17222919 in IS and control subjects.

Population 1	rs17222919	IS subjects (n = 910, n (%))	Control subjects (n = 925, n (%))	*P* value	Adjusted OR (95% CI)
	TT	593 (65.2)	550 (59.5)		1.000
	TG	288 (31.6)	327 (35.3)	0.043*	0.817 (0.671–0.994)
	GG	29 (3.2)	48 (5.2)	0.016*	0.560 (0.348–0.901)
	T allele	1474 (81.0)	1427 (77.1)		1.000
	G allele	346(19.0)	423 (22.9)	0.004*	0.792(0.675–0.929)
Population 2	rs17222919	IS subjects (n = 1003, n (%))	Control subjects (n = 889, n (%))	*P* value	Adjusted OR (95% CI)
	TT	658 (65.6)	529 (59.5)		1.000
	TG	312 (31.1)	313 (35.2)	0.025*	0.801 (0.660–0.973)
	GG	33 (3.3)	47 (5.3)	0.014*	0.564 (0.356–0.894)
	T allele	1628 (81.2)	1371 (77.1)		1.000
	G allele	378(18.8)	407 (22.9)	0.002*	0.782 (0.668–0.915)

*P* value and OR (95% CI) were adjusted for confounding factors.

* express the adjusted *P* value for significance *P*<0.05

To assess the effect of rs17222919 on the risk of IS, we compared additive, dominant, and recessive models. The effect of rs17222919 was best described with an additive model (Table 3).

**Table 3:** Detailed association of rs17222919 between IS and control groups under different genetic models.

	Model	Genotype	Case (n, %)	Control (n, %)		χ^2^	*P* value	OR(95%CI)
Population 1	Dominant	TG+GG	317(34.8)	375(40.5)				
		TT	593(65.2)	550(59.5)	6.357		0.012	1.275(1.056–1.541)
	Recessive	GG	29(3.2)	48(5.2)				
		TT+TG	881(96.8)	877(94.8)	4.575		0.032	1.663(1.039–2.661)
	Additive	TT	593(65.2)	550(59.5)				
		TG	288(31.6)	327(35.3)	4.082		0.043*	0.817 (0.671–0.994)
		GG	29(3.2)	48(5.2)	5.836		0.016*	0.560 (0.348–0.901)
Population 2	Dominant	TG+GG	345(34.4)	360(40.5)				
		TT	658(65.6)	529(59.5)	7.497		0.006	1.298(1.077–1.565)
	Recessive	GG	33(3.3)	47(5.3)				
		TT+TG	970(96.7)	842(94.7)	4.640		0.031	1.641(1.041–2.585)
	Additive	TT	658(65.6)	529(59.5)				
		TG	312(31.1)	313(35.2)	5.004		0.025*	0.801 (0.660–0.973)
		GG	33(3.3)	47(5.3)	6.081		0.014*	0.564 (0.356–0.894)

*P* value and OR (95% CI) were adjusted for confounding factors.

* express the adjusted *P* value for significance *P*<0.05

To further study the association of rs17222919 with IS in different sexes, we compared the frequency of the rs17222919 genotype between case and control within sexes in Population 1. In males, the rs17222919 GG genotype frequency was significantly different between case and control groups (2.1% and 5.2% respectively) (Table 4). The rs17222919 TG genotype and G allele frequencies were significantly lower in the female IS group than the female control group (*P*<0.05) (Table 4).

**Table 4:** Stratified analysis of the relationship between rs17222919 Genotypes and susceptibility of IS.

Population 1	Case (n, %)	Control (n, %)	*P* value	OR(95%CI)	Population 2	Case (n, %)	Control (n, %)	*P* value	OR(95%CI)
male	n = 479	n = 478			Male	n = 542	n = 458		
TT	308(64.3)	302(63.2)			TT	352(64.9)	288(62.9)		
TG	161(33.6)	151(31.6)	0.750	1.045(0.796–1.374)	TG	177(32.7)	147(32.1)	0.913	0.985(0.753–1.289)
GG	10(2.1)	25(5.2)	0.012*	0.392(0.185–0.831)	GG	13(2.4)	23(5.0)	0.027*	0.462(0.230–0.929)
T allele	777(81.1)	755(79.0)			T	881(81.3)	723(78.9)		
G allele	181(18.9)	201(21.0)	0.243	0.875(0.699–1.095)	G	203(18.7)	193(21.1)	0.190	0.863(0.693–1.076)
female	n = 431	n = 447			Female	n = 461	n = 431		
TT	285(66.1)	248(55.5)			TT	307(66.6)	238(55.2)		
TG	127(29.5)	176(39.4)	0.001*	0.628(0.472–0.835)	TG	134(29.1)	172(39.9)	0.0004*	0.604(0.455–0.801)
GG	19(4.4)	23(5.1)	0.303	0.719(0.382–1.351)	GG	20(4.3)	24(4.9)	0.348	0.738(0.391–1.394)
T allele	697(80.9)	672(75.2)			T	748 (81.1)	648 (75.2)		
G allele	165(19.1)	222(24.8)	0.004*	0.717(0.571–0.900)	G	174 (18.9)	214 (24.8)	0.002*	0.704(0.562–0.883)

*P* value and OR (95% CI) were adjusted for confounding factors.

* express the adjusted *P* value for significance *P*<0.05

After adjustment for cardiovascular risk factors, potential associations between rs17222919 variants and stroke subtypes defined by TOAST were shown in Table 5. In Population 1, subgroup analysis indicated that this association was limited to cases with LAA (OR = 0.815, 95% CI = 0.687–0.967, *P* = 0.019) and SUE (OR = 0.661, 95% CI = 0.459–0.951, *P* = 0.025), but not to cases with SAO (OR = 0.786, 95% CI = 0.533–1.159, *P* = 0.223).

**Table 5:** Genotype and allelic distribution of rs17222919 in IS subtypes and controls.

Population 1	Control(n = 925)	Patients with ischemic stroke		
		LAA(n = 704)	SA0(n = 90)	SUE(n = 116)
TT	550(59.5)	450(63.9)	60(66.7)	83(71.6)
TG	327(35.4)	234(33.2)	26(28.9)	28(24.1)
GG	48(5.1)	20(2.9)	4(4.4)	5(4.3)
OR(95%CI),TT vs TG	1.00	0.875(0.710–1.078)	0.729(0.451–1.178)	0.567(0.362–0.890)
P value	1.00	0.209	0.195	0.013*
OR(95%CI),TT vs GG	1.00	0.509(0.298–0.871)	0.764(0.266–2.192)	0.690(0.267–1.784)
P value	1.00	0.012*	0.616	0.441
G allele (%)	22.9	19.5	18.9	16.4
P value	1.00	0.019*	0.223	0.025*
OR(95%CI)	1.00	0.815(0.687–0.967)	0.786(0.533–1.159)	0.661(0.459–0.951)
Population 2	Control(n = 889)	LAA(n = 675)	SAA(n = 198)	SUE(n = 130)
TT	529(59.5)	431(63.9)	133(67.2)	94(72.3)
TG	313(35.2)	225(33.3)	56(28.3)	31(23.8)
GG	47(5.3)	19(2.8)	9(4.5)	5(3.9)
OR(95%CI),TT vs TG	1.00	0.882(0.713–1.092)	0.712(0.505–1.002)	0.557(0.363–0.856)
P value	1.00	0.250	0.051	0.007*
OR(95%CI),TT vs GG	1.00	0.496(0.287–0.858)	0.762(0.364–1.593)	0.599(0.232–1.544)
P value	1.00	0.011*	0.468	0.284
G allele (%)	22.9	19.5	18.7	15.8
P value	1.00	0.021*	0.068	0.010*
OR(95%CI)	1.00	0.815(0.685–0.970)	0.774(0.588–1.020)	0.631(0.444–0.896)

*P* value and OR (95% CI) were adjusted for confounding factors.

* express the adjusted *P* value for significance *P*<0.05

### ***ALOX5AP*** promoter activity analysis

**Fig 1:**
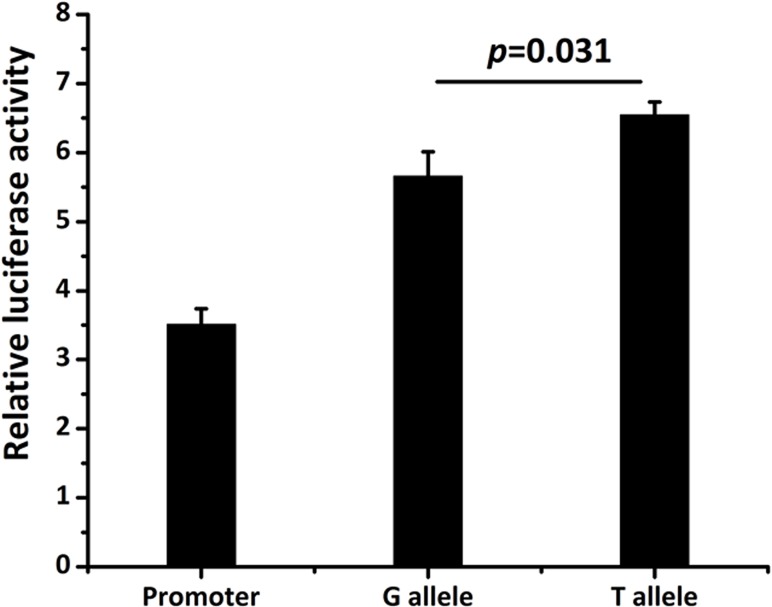
Effects of the promoter Polymorphism (rs17222919, −1316T/G) of ALOX5AP gene on transcriptional activity. The transcription activity was measured using an *in vitro* luciferase assay and the results were shown as the means ± SE values. Data were expressed as the fold-increase in luciferase activity relative to pGL3- promoter. A one-way ANOVA was used to assess statistical significance.

We next investigated whether the polymorphism at position −1316 affected the transcriptional activity of the *ALOX5AP* promoter in HEK293 cells. The experiments showed that significantly lower levels of luciferase were produced by the G allele construct compared with the T allele construct (*P* = 0.031, Fig 1), although these levels were higher than the pGL3-promoter reference vector (*P* = 0.000). This suggested that the G allele has a lower transcription activity than the T allele.

### Replication study

The effects seen in Population 1 with the rs17222919 variants were next analyzed in Population 2 from the southern Henan Province. Similar to the first population, we found that both GG genotype and G allele frequencies in LAA and SUE subtypes of Population 2 were significantly lower than those in the control group after adjusting for conventional risk factors (*P*<0.05, Table 5). Rs17222919 was also associated with IS in an additive genetic models (Table 3). In males, the rs17222919 GG genotype frequency was significantly different between case and control (*P*<0.05). Whereas in females, the TG genotype and G allele frequencies were significantly lower in the IS group than in control (Table 4). Finally, functional analysis supported a lower transcription activity for allele G than allele T, as seen in Population 1. Together, these results suggested that rs17222919 was associated with IS in Population 2.

## Discussion

In the present study, we designed a two-stage study to explore the relationship between rs17222919 and IS risk in a central Chinese Han population (S2 and S3 Datasets). Our findings showed that this common functional variation in the *ALOX5AP* promoter region was significantly associated with a reduced risk of IS in two independent Chinese cohorts (*P* = 0.004, OR = 0.792, 95% CI = 0.675–0.929; *P* = 0.002, OR = 0.782, 95% CI = 0.668–0.915). To the best of our knowledge, this is a comprehensive study to evaluate whether the rs17222919 polymorphism could influence susceptibility to IS in a large Han Chinese population. The result verified our hypothesis that there might be some unidentified variants in the *ALOX5AP* promoter region associated with the potential risk of IS.

A major strength of the present study was the design of two independent cohorts, which greatly reduced the possibility of identifying a false-positive finding from the genetic association study [17]. For the rs17222919 polymorphism, GG/GT genotype frequencies and G allele frequencies of IS groups were significantly lower than those of control groups in both independent populations (*P* <0.05). Multivariate logistic regression analysis showed that the GG genotype was associated with a 0.560-fold increased risk of IS after adjusting for conventional risk factors (95% CI, 0.348–0.901; *P* = 0.016). Next, genotype association test with dominant, recessive and additive models were performed and rs17222919 was associated with IS in an additive genetic model in both cohorts (*P* <0.05). However, this association was inconsistent with the results reported previously by Kim et al, who showed that the rs17222919 was associated with intracerebral hemorrhage, but not IS, in a Korean population. [13] Moreover, our results were so different from Wang *et al*, who reported that the rs17222919 G allele was associated with an increased risk of IS, especially with the small vessel subtype in an eastern Chinese Han population[18]. The controversial results of rs17222919 between us may be attributed to the differences in ethnic background, sample size, statistical analysis and so on. Therefore, further study with different population is required to confirm the findings.

After stratifying our data by sex and subtype, we detected significant differences in the TG genotype and G allele frequency distribution of rs17222919 between female IS patients and female controls. The minor G allele was revealed to be associated with a decreased risk of IS in females. These findings indicated that the rs17222919 G allele was the protective allele against the development of IS. In addition, the rs17222919 GG genotype was associated with a decreased risk of IS in males. Subgroup analysis indicated that this association was limited to cases with LAA (OR = 0.815, 95% CI = 0.687–0.967, *P* = 0.019) and SUE (OR = 0.661, 95% CI = 0.459–0.951, *P* = 0.025), but not to cases with SAO (OR = 0.786, 95% CI = 0.533–1.159, *P* = 0.223). The difference between sex, subtype and the association with IS may be attributed to the complex etiology and multifactorial background of IS pathogenesis.

Promoter elements have primary roles in regulating gene transcription. Altering the DNA sequence may result in changes in transcription factor binding sites or binding speeds. Thus, a genetic variant in a promoter element can affect gene transcription and therefore gene function [19]. Another important finding of the present study was proved that the rs17222919 T>G was the functional causative SNP for the associations with IS. Because rs17222919 T>G is located in the *ALOX5AP* promoter region, we investigated whether it could modulate promoter activity using an established luciferase assay. This *in vitro* promoter assay revealed that the G allele had a lower transcription activity than the T allele, suggesting that the −1316T/G variation reduces *ALOX5AP* promoter activity and consequently down-regulation of gene expression. This finding differed from the results of Antonio et al, who reported increased luciferase production from the G allele construct compared with the T allele construct[20]. However, our results can be supported by the evidence that the glucocorticoid receptor binds to T-containing sequences, but that no transcription factor binds to G-containing sequences in the rs17222919 site [13]. Therefore, additional studies should be conducted to confirm our results. Based on these preliminary results, we propose a possible molecular mechanism for the role of rs17222919 in the decreased risk of IS as follows: first, the G nucleotide reduces the binding activity of the locus, which might down-regulate *ALOX5AP* transcription by altering the glucocorticoid receptor binding site through certain mechanisms. Second, the decreased *ALOX5AP* transcription results in increased inactivation of the 5-LO pathway and reduces biosynthesis of leukotrienes, which in turn protects from IS risk.

Limitations of the present study include the fact that *ALOX5AP* mRNA levels were not compared between cases and controls, which would have confirmed the results of the luciferase assay. Additionally, we did not test LTB_4_ plasma levels (a key product of the FLAP/5-LO pathway) in different rs17222919 genotypes both in the IS and control groups.

In conclusion, we demonstrated that the rs17222919 polymorphism is associated with a decreased risk of IS in two independent Chinese populations. More importantly, an *in vitro* promoter assay revealed that the rs17222919 G allele has a lower transcription activity than the T allele, suggesting that the −1316T/G variation in the *ALOX5AP* promoter region could reduce promoter activity and down-regulate gene expression. Future studies on the additional variants in the *ALOX5AP* promoter and their biological function should be conducted to determine the etiology of IS.

## Supporting Information

S1 Fig The allelic discrimination plot of rs17222919 polymorphism TaqMan genotyping. (TIF)

S2 Fig The DNA reverse sequencing chromatogram of rs17222919 polymorphism (GG/GT). (TIF)

S1 Dataset Luciferase assay data. (XLS)

S2 Dataset First population data. (XLSX)

S3 Dataset Replication population data. (XLSX)
