# Supplementary figures and images for: A Promoter Polymorphism (rs17222919, −1316T/G) of ALOX5AP Gene Is Associated with Decreased Risk of Ischemic Stroke in Two Independent Chinese Populations
Source: PLoS One. 2015 Mar 27;10(3):e0122393. doi: 10.1371/journal.pone.0122393 (PMC4376390; doi:10.1371/journal.pone.0122393)

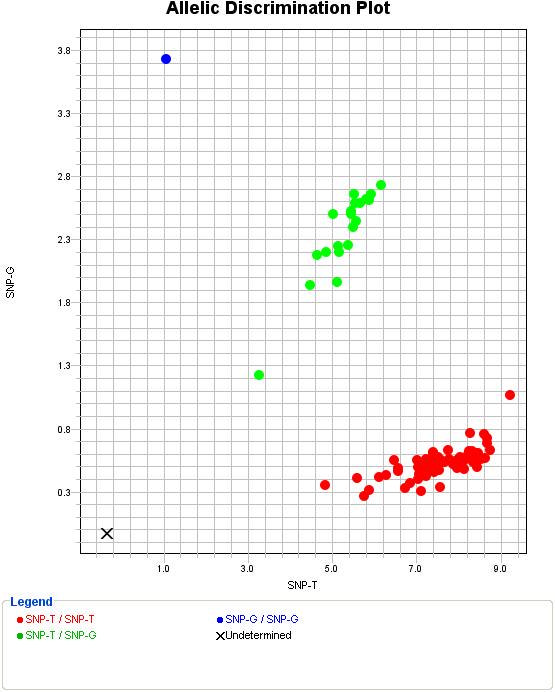

Supplement: S1 Fig — (TIF) [file pone.0122393.s001.tif]

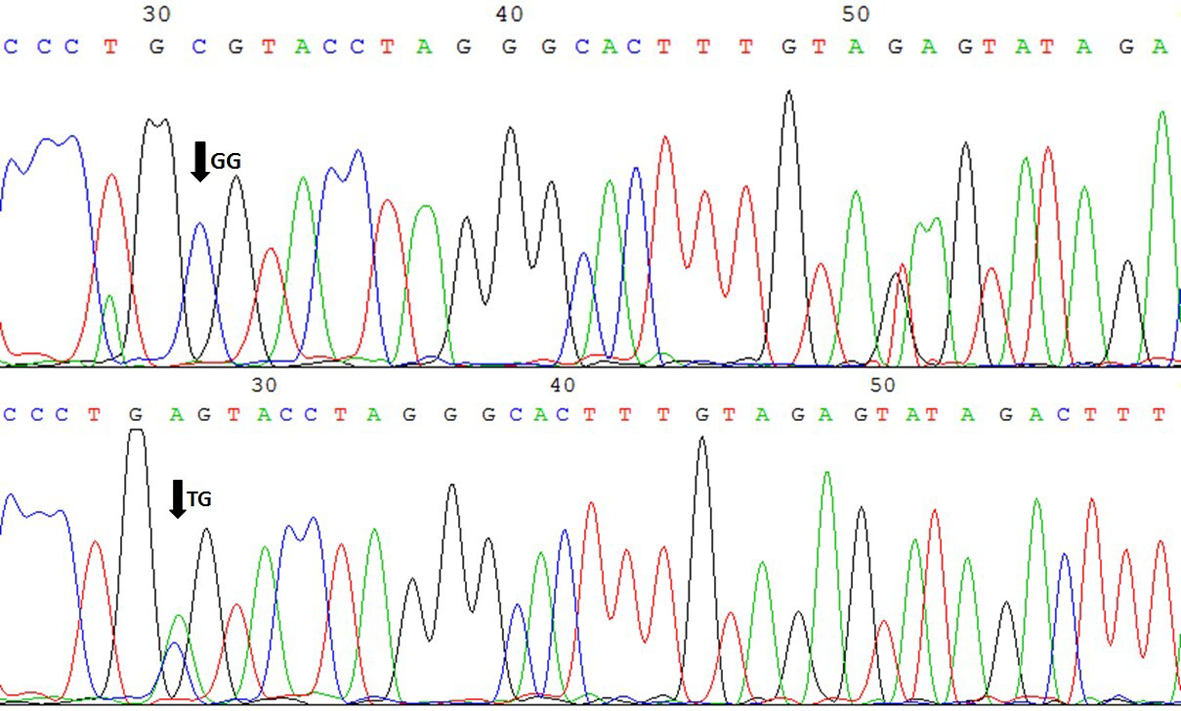

Supplement: S2 Fig — (TIF) [file pone.0122393.s002.tif]
